# Advances and Perspectives in the Treatment of B-Cell Malignancies

**DOI:** 10.3390/cancers13092266

**Published:** 2021-05-08

**Authors:** Marta Cuenca, Victor Peperzak

**Affiliations:** Center for Translational Immunology, University Medical Center Utrecht, Utrecht University, 3584 CX Utrecht, The Netherlands; m.cuenca@umcutrecht.nl

B-cell malignancies arise from different stages of B-cell differentiation and constitute a heterogeneous group of cancers including B-cell lymphomas, B-cell leukemias, and plasma cell dyscrasias. Within each subgroup, intra- and inter-patient heterogeneity can greatly influence the clinical course of the disease and consequently, treatment choice. Basic and translational research efforts worldwide are focused on tackling the remaining challenges in the treatment of these hematological disorders, including (i) strategies to (further) improve immunotherapeutic approaches, (ii) the search for novel (metabolic) targets in malignant cells, (iii) understanding mechanisms of resistance to therapy, and (iv) feasible strategies to personalize treatment. This Special Issue of *Cancers* includes eighteen articles (three original papers and fifteen reviews) by international leaders in the field of B-cell malignancies, covering the latest findings related to each of these challenges.

During the last years, novel immunotherapeutic approaches, such as chimeric antigen receptor (CAR) T-cell therapy and immune checkpoint inhibitors (ICIs), have shown potential for improving the outcomes of patients with refractory B-cell malignancies. The success of immunotherapy, however, is dampened by inhibitory signals or pathways that are active in malignant cells itself or that are derived from the tumor microenvironment (TME). This reduces the anti-tumor activity of the immune system, particularly of T cells. Montironi and colleagues describe the similarities and differences of T-cell dysfunctionality in solid versus B-cell malignancies, with a special focus on immunometabolism. The authors map soluble and cognate factors conditioning the immunosuppressive nature of the TME across different cancer types and the mechanisms by which the tumor niche impairs T-cell (metabolic) activity, suggesting that armoring T cells with enhanced metabolic functions could increase immunotherapy efficacy [1]. 

In line with this topic, van Bruggen et al. provide an overview of how the multiple levels of T-cell dysfunction in B-cell non-Hodgkin lymphoma (B-NHL) could serve as novel targets to improve the efficacy of T-cell-based therapies. The authors integrate clinical data from trials using ICIs, bispecific antibodies, and CAR-T cells with T-cell alterations observed in B-NHL patients. The authors list possible solutions to overcome T-cell dysfunction (such as exhaustion and skewing or the expansion of regulatory T cells), including combination therapies with immunomodulatory drugs (IMIDs) or improvement of CAR-T-cell manufacturing to maximize their persistence [2]. Alternatively, strategies engaging innate immune cells to fight B-cell malignancies represent an attractive and rapidly evolving field. In the context of monoclonal antibody (mAb) therapy, van der Horst and colleagues describe different Fc-engineering strategies to enhance antibody-dependent cellular cytotoxicity (ADCC), antibody-dependent cellular phagocytosis (ADCP), and complement-dependent cytotoxicity (CDC), with an overview of the (pre-)clinical status of Fc-engineered mAbs in chronic lymphocytic leukemia (CLL), B-NHL, and multiple myeloma (MM) [3]. 

In the last decade, the number of clinical trials addressing immune checkpoint-based therapies in B-cell lymphoma has increased significantly. Armengol et al. provide an exhaustive overview of the current arsenal of ICIs used in B-cell lymphomas, detailing their clinical evaluation and reported adverse events. The authors highlight the potential of combination immunotherapy involving PD-1/PD-L1 and CTLA-4 inhibitors, and anticipate the implementation of biomarker-driven trials where patient-specific profiling of cell subtypes and genomic alterations in the TME will identify optimal targets [4]. A well-established mechanism by which malignant B cells can evade anti-tumor immune responses is the overexpression of the oncogene MYC. De Jonge et al. review how MYC overexpression dampens adaptive and innate immunity against aggressive B-NHL, influencing tumor cell metabolism and interplay with the TME. The authors summarize clinical and pre-clinical data regarding the efficacy of MYC modulators in lymphoid malignancies, stating that beneficial effects of MYC downregulation in malignant target cells may be overshadowed by the effects of MYC downregulation in immune effector cells, which compromises their function. Clinical outcomes of diffuse large B-cell lymphoma (DLBCL) patients treated with immunotherapy indicate that cellular immunotherapy seems the most promising option for patients harboring MYC overexpression [5]. Malignant B cells can also avoid immune surveillance via advantageous mutations that can remodel the TME and inactivate leukocytes; this is a hallmark of follicular lymphoma (FL). Dobaño-López and colleagues review how the mutational landscape of FL influences its interplay with the (permissive) microenvironment, and summarize current therapies targeting the FL–TME crosstalk, including ICIs, B-cell receptor (BCR) inhibitors, and metabolic regulators [6]. In classical Hodgkin lymphoma (cHL), thymus and activation-regulated chemokine (TARC) represents a canonical example of a cancer cell-expressed molecule which is both critical for tumor–TME interactions and usable as a biomarker. Zijtregtop et al. describe the essential role of TARC in Hodgkin and Reed–Sternberg (HRS) cell survival and the recruitment of Th2 cells, highlighting its clinical applicability as a diagnostic biomarker—in oncological diseases and beyond—and how TARC inhibition could potentially improve the outcome of cHL patients [7]. 

Metabolic pathways in cancer cells are often intensely reprogrammed as a result of oncogene activation. Bloedjes et al. review how recurrent chromosomal aberrations in MM drive the activation of specific oncogenes, profoundly altering the metabolic state of malignant plasma cells, and uncovering dependencies that may be targeted therapeutically [8]. In line with this, Bordini and colleagues show that dysregulated iron homeostasis increases MM cell susceptibility to proteasome inhibitors. Thus, high-dose iron causes the accumulation of polyubiquitinated proteins and improves the killing efficacy of carfilzomib and bortezomib in an in vivo MM model (Vk*MYC mice) [9]. The over-production of proteins in MM cells renders them highly sensitive to drugs modulating the ubiquitin–proteasome system (UPS), such as proteasome inhibitors and thalidomide analogs. Wirth et al. review the biology and clinical development of small-molecule UPS modulators, and how they can be exploited for MM treatment [10]. Alterations in the intrinsic apoptosis pathway are also frequently observed in MM, CLL, and NHL. Consequently, cancer cell dependence on pro-survival proteins represents a targetable weakness. Venetoclax, a selective BCL-2 inhibitor, has shown clinical efficacy in CLL and acute myeloid leukemia (AML). Lin et al. review recent (pre-)clinical data regarding the use of BCL-2, MCL-1, and BCL-XL inhibitors for the treatment of B-cell malignancies, and possible mechanisms of resistance associated with these compounds [11]. 

Primary and acquired resistance to therapy often poses a major challenge in the management of B-cell malignancies. George et al. provide an overview of (non-)genetic and TME-related factors driving resistance to ibrutinib in B-cell lymphomas. The authors describe the efficacy of several therapeutic strategies for treating ibrutinib-refractory lymphoma patients, including new-generation BTK inhibitors, proteolysis-targeting chimeras (BTK-PROTACs), and CD19-directed CAR-T cells [12]. In turn, Berendsen et al. review the genetic alterations enriched in relapsed or refractory (R/R) DLBCL, elaborating on how pathways affected by these mutations could potentially mediate drug resistance. The authors summarize recent clinical findings regarding the therapeutic targeting of relapse-associated drivers (including the BCL-2 family members, MYC and p53), which suggest that combinations of targeted therapies may lead to synergizing effects and be beneficial for treating R/R DLBCL patients [13]. In connection with how genetic alterations can promote lymphomagenesis in DLBCL, Priebe et al. show that ETS1 overexpression, associated with ch11q24.3 gain, contributes to the molecular pathogenesis of ABC-DLBCL regulating B-cell activation, proliferation, and resistance to apoptosis. The authors identify the Fc receptor for IgM (FCMR) as a novel ETS1 target overexpressed in ABC-DLBCL [14]. Further exploring molecular factors regulating B-NHL growth, Niu et al. demonstrate that mir-26b-5p inhibits Burkitt lymphoma cell proliferation and its effect is mediated, at least in part, by binding to KPNA2 [15]. 

The expanding therapeutic arsenal for treating B-cell malignancies comes hand in hand with the possibility of—and the need for—personalizing treatment strategies. Multiple myeloma (MM) is no exception, with more than 20 different approved drugs and several possible combination regimens. Cazaubiel and colleagues review the current approaches that are being explored to adapt treatment strategies in MM considering, besides patient age and clinical disease presentation, a revised definition of high-risk groups and the presence of minimal residual disease (MRD). The authors provide an overview of recent and prospective clinical trials evaluating the strength of MRD status not only as a prognostic factor, but also as a tool to adapt therapy duration and even identify groups of patients that would benefit from treatment-free monitoring [16]. This Special Issue also includes reviews on uncommon clinical entities such as primary cutaneous and primary pulmonary B-cell lymphomas [17,18], describing their clinical and pathological characteristics, as well as current therapeutic options.

In conclusion, this Special Issue provides updated knowledge concerning the development of novel treatment options, including targeted therapies, combination treatment, and immunotherapeutic approaches that cover basic, translational, and (pre-)clinical aspects aiming to improve treatment of B-cell malignancies.
